# Sublingual Fast-Dissolving Thin Films of Loratadine: Characterization, In Vitro and Ex Vivo Evaluation

**DOI:** 10.3390/polym16202919

**Published:** 2024-10-17

**Authors:** Yahya Alhamhoom, Ashitha Kakarlapudi Said, Avichal Kumar, Shivakumar Hagalavadi Nanjappa, Divya Wali, Mohamed Rahamathulla, Syeda Ayesha Farhana, Mohammed Muqtader Ahmed, Thippeswamy Boreddy Shivanandappa

**Affiliations:** 1 Department of Pharmaceutics, College of Pharmacy, King Khalid University, Al Faraa, Abha 62223, Saudi Arabia; ysalhamhoom@kku.edu.sa (Y.A.); rahapharm@gmail.com (M.R.); 2 Department of Pharmaceutics, KLE College of Pharmacy, Rajajinagar, Bengaluru 560010, India; ashithavarma167@gmail.com (A.K.S.); avichalk0994@gmail.com (A.K.); divyawali28@gmail.com (D.W.); 3 Department of Pharmaceutics, College of Pharmacy, Qassim University, Buraidah 51452, Saudi Arabia; a.farhana@qu.edu.sa; 4 Department of Pharmaceutics, College of Pharmacy, Prince Sattam Bin Abdul Aziz University, Al Kharj 11942, Saudi Arabia; muqtadernano@psau.edu.sa; 5 Department of Biomedical Science, College of Pharmacy, Shaqra University, Al-Dawadmi Campus, Al-Dawadmi 11961, Saudi Arabia

**Keywords:** loratadine sublingual thin film, hydroxypropyl methylcellulose (E15), polyvinyl pyrrolidone (K30), mucoadhesion, in vitro study, bioavailability

## Abstract

Loratadine (LOR) is a second-generation antihistamine that exhibits a low and variable oral bioavailability (10–40%) and delayed onset owing to poor solubility and an extensive first-pass effect. Therefore, in light of the clinical need, the main goal of the present study was to develop sublingual fast-dissolving thin films of LOR–citric acid co-amorphous systems (LOR-CAs) with the aim of eliciting a faster onset and improving the bioavailability. We formulated sublingual fast-dissolving thin films of LOR by a film-casting technique using hydrophilic polymers like hydroxypropyl methylcellulose (HPMC E15), polyvinyl pyrrolidone K30 (PVP K30), and hydroxypropyl cellulose EL (HPC-EF) and citric acid as a pH modulator, while glycerin served as a plasticizer. The sublingual fast-dissolving thin films were characterized by FTIR, SEM, DSC, and XRD and evaluated for in vitro dissolution and ex vivo mucoadhesion. The best formulation (F1) developed using HPMC E15 as a polymer, glycerin as a plasticizer, and citric acid as a pH modulator was found to be the optimized formulation as it was smooth, clear, flexible, and displayed good mucoadhesion (11.27 ± 0.418 gm/cm^2^) and uniform thickness (0.25 ± 0.02 mm). The formulation F1 was found to display a significantly shorter DT (30.30 ± 0.6 s) and rapid release of LOR (92.10 ± 2.3% in 60 min) compared to other formulations (ANOVA, *p* < 0.001). The results indicated that the prepared sublingual films are likely to elicit a faster therapeutic effect, avoid first-pass metabolism, and improve the bioavailability.

## 1. Introduction

The oral route is the most preferred mode of administration of drugs as it is safe, convenient, and economical. Although the oral route is most convenient for drug administration, bioavailability is always a challenge with conventional dosage forms due to poor solubility or an extensive first-pass metabolism effect [1]. Numerous approaches have been employed to improve the oral bioavailability of poorly soluble drugs and extensively metabolizable therapeutic agents. Although novel drug delivery systems have been successful in addressing these issues, newer platform technologies are needed to develop simple oral dosage forms that could be easily scaled-up and commercialized [2]. Allergy is a reaction by the immune system that is known to affect nearly 10 to 30% of the worldwide population. The high prevalence of allergic diseases has been a major health care issue that remains unexplained [3]. The range of allergic diseases includes rhinitis, sinusitis, and asthma, which negatively impact the quality of life of those affected. Loratadine (LOR) is a powerful H1 antagonist commonly used in the treatment of various allergic conditions like rhinitis and urticaria. LOR is metabolized in the liver by the cytochrome P450 enzymes CYP3A4 and CYP2D6 resulting in the formation of the active metabolite desloratadine [4]. Desloratadine has a longer half-life of 27 h contributing to the prolonged antihistamine effects of loratadine [5]. LOR is considered advantageous as it is devoid of sedative effects and displays low toxicity compared to other anti-allergic drugs [4]. According to findings from controlled clinical studies, the recommended daily dose of LOR is 10 mg for adults and children over 12 years [5]. The drug is a weak base belonging to the biopharmaceutical classification system (BCS) Class IIb as it exhibits low solubility but high permeability [6,7]. In an acidic environment (stomach pH 1.5 to 3.5), LOR exhibits slightly increased solubility due to its basic nature [8]. Therefore, LOR becomes more ionized in acidic conditions, which further enhances its solubility compared to neutral or alkaline environments (~0.005 mg/mL) that would result in poor bioavailability (10–40%) [9]. The poor and variable bioavailability of LOR can be attributed to extensive first-pass metabolism too [10]. To resolve the bioavailability issues, a number of attempts have been made to develop nanoparticles [6], buccoadhesive wafers [11], oral films with nanoparticles [12,13], and oral films containing micronized LOR [14]. These preparations were developed considering the fact that LOR would only be absorbed after it reaches the intestine delaying the onset to nearly 75–180 min for LOR [15]. In this context, sublingual tablets of LOR have been proposed for the management of various allergic conditions considering the need to elicit a quicker response [16]. However, the disintegration of these tablets would be a rate-limiting step for drug release in salivary pH.

In this context, we plan to develop sublingual fast-dissolving thin films of LOR citric acid co-amorphous systems (LOR-CAs) that are known to quickly disintegrate and release the content in the sublingual region to elicit a faster onset. Co-amorphous systems are a uniform blend of a drug with low molecular weight carriers termed co-formers that can stabilize the drug [17]. Several medications of LOR are available on the market under the brand names of Claritin^®^ (regular and dissolvable tablets), Alavert^®^ (quick-dissolving tablets), and Dimetapp^®^ (specifically for children) that are meant to offer quick relief from allergy symptoms [18]. As far as our knowledge goes, there are no reports that have attempted to mitigate the pH-dependent solubility of LOR in the development of sublingual fast-dissolving thin films employing the proposed strategy. Sublingual thin films are known to elicit a faster onset owing to the thinner epithelium and high vasculature of the sublingual mucosa. The oral cavity has offered a promising platform for formulators due to the high permeability of the mucus membrane [19]. Palatability and mouth feel are the key advantages of orodispersible films that can be used to deliver potent molecules [20]. In addition to evading the first-pass effect, the sublingual route offers the additional advantage that it is able to elicit a faster onset of action [21].

Considering these factors, the present work aims to produce sublingual fast-dissolving thin films of LOR using different proportions of hydrophilic bioadhesive polymers such as hydroxypropyl methyl cellulose E15 (HPMC E15). Other polymers like polyvinyl pyrrolidone K30 (PVP K30) and hydroxypropyl cellulose EL (HPC-EF) are also utilized for film formulation. Further, citric acid is used as a pH modulator and glycerin as a plasticizer. The prepared sublingual fast-dissolving thin films are characterized using Fourier transform infrared (FTIR) spectroscopy, scanning electron microscopy (SEM), X-ray diffractometry (XRD), and differential scanning calorimetry (DSC). In addition, the films are evaluated for in vitro dissolution studies, ex vivo mucoadhesion, and stability studies and finally subjected to statistical analysis.

## 2. Materials and Methods

### 2.1. Materials

Loratadine (LOR) with ≥99.00% purity was obtained from Vasudha Chem Limited, Vishakhapatnam, India; hydroxypropyl methyl cellulose (HPMC E15); polyvinyl pyrrolidone K30 (PVP K30); hydroxypropyl cellulose EF (HPC-EF); citric acid; sodium saccharin; glycerin; peppermint flavor and ethanol with the purity range of 97–99% were purchased from S.D. Fine Chemicals, Mumbai, India. Porcine gastric mucin (PGM), alpha-amylase from porcine pancreas, Tween 20^®^, and xanthan gum were sourced from Sigma-Aldrich, Dorset, UK. All additional chemicals and solvents used in the dissolution experiments were obtained from Fisher Scientific, Loughborough, UK.

### 2.2. Formulation of Sublingual Fast-Dissolving Thin Films

Film-forming hydrophilic polymers, including HPMC E15, HPC-EF, and PVP K30, were used for the formulation of sublingual thin films of LOR by the film-casting technique [22]. Casting solutions composed of polymeric solutions were prepared by dissolving the required quantity of polymer, plasticizer, and saliva-stimulating agent in ethanol on a magnetic stirrer at room temperature (REMI Magnetic Stirrer, 2 ML, Remi Elektrotechnik Limited, Mumbai, India). Then the drug was added to the above solution and allowed to stir on a magnetic stirrer for 30 min to ensure complete dissolution [22]. Finally, sweetening and flavoring agents were added to the polymeric solutions containing LOR-CA. The air bubbles formed during the process were removed by ultra sonication (GT SONIC, AN-SS-3L, Shenzhen, China), and the clear casting solutions measuring 10 mL were poured into the Petri plate, which had an area of 12.6 cm^2^ [23]. The plate was placed under a vacuum oven (Servewell Instruments, Karnataka, India) under controlled pressure (300 mm·Hg) and temperature (25 ± 2 °C) overnight to ensure complete removal of the solvent [24]. The composition of the casting solutions is indicated in Table 1. Every single formulation of the film was repeated three times to check the reproducibility of the proposed compositions. The films casted were stored in airtight aluminium self-sealable pouches and stored in a desiccator (BR65805, Sigma-Aldrich, Shanghai, China) at room temperature (25 ± 2 °C).

### 2.3. Evaluation of Sublingual Fast-Dissolving Thin Films

#### 2.3.1. Morphological and Surface Topographical Analysis

SEM photographs of the appropriate resolution were acquired using a scanning electron microscope (SEM FEI Quanta 200F, Hillsboro, OR, USA). The morphology and surface topography of oral films are usually assessed by SEM [25]. The samples were mounted on an SEM sample stab using double-sided sticking tape and gold-coated (~200 nm) under reduced pressure (0.0133 Pa) for 5 min using an ion sputtering device at 10 kV energy using an EHT detector. Bright field microscopy (Labomed Vision 2000, Mumbai, India) was also performed to assess the morphology of the casted films. The casted films were mounted on the glass slide and illuminated under a bright light source. The samples were examined under a magnification of 40× to capture the surface morphology of the films [26].

#### 2.3.2. Drug–Polymer Compatibility Analysis

Fourier transform infrared (FTIR) spectrometry was used to assess the compatibility between LOR and the other excipients used in the optimized film [27]. The LOR and polymers were physically mixed and stored at ambient temperature for one month before analysis. Then, the sample was mixed with potassium bromide and loaded into a diffuse reflectance sample holder before exposure to infrared radiation [28]. The data were acquired in the scanning range of 600–4000 cm^−1^ in an FTIR spectrophotometer (Jasco 450 Plus spectrophotometer, ECC 450, Easton, MD, USA).

#### 2.3.3. Physicochemical Characterization of Sublingual Fast-Dissolving Thin films

##### Weight Variation

Samples collected from three different areas of the casted sample films measuring 1 × 1 cm^2^ were individually weighed on an electronic balance (Analytical Balance, Shimadzu BL-220H, Kyoto, Japan) [29]. The average weight of the selected films was determined before expressing the results as the mean ± standard deviation (SD) of three determinations.

##### Thickness Measurement

Samples from each casted film formulation were selected from three different regions of the film to determine the thickness. Digital calipers (Mitutoyo 500-196-30 Digital Caliper, Kawasaki, Japan) was used to determine the thickness of the randomly selected films [30]. The results were expressed as the mean ± SD of three determinations.

##### Folding Endurance

The folding endurance (FE) of sublingual thin films was assessed by repeatedly folding each film at the same point until it broke. The FE was estimated by considering the number of folds made at the same point without the film breaking [31]. Three measurements were performed with three different samples of the same formulation and the results were expressed as the mean ± SD.

##### Tack Test

Tackiness indicates the stickiness or adhesion of the polymeric films produced and can be performed with dry film as well as retaining a certain amount of moisture [32]. Generally, bioadhesive polymeric films exhibit good adhesion. The tackiness of the film was generally determined by applying pressure manually using the thumb [33]. Three trials for each casted film were performed to check the repeatability of the results obtained.

##### Surface pH

The term surface pH refers to the pH at the surface of a film, especially relevant in formulations like fast-dissolving oral films used in drug delivery systems [34]. Surface pH is a critical parameter as it determines the irritation potential of the sublingual film. The pH of the films was recorded by taking them in the Petri dish containing 0.5 mL of deionized water. The film was soaked and equilibration with deionized water for ~40 s. After the equilibration, the pH meter electrode was brought into contact with the film surface to record the pH value using a pH meter (Digital pH Meter 7007, Digisun Electronics, Hyderabad, Indian) at 37 °C ± 2 °C [34]. The results of the surface pH were expressed as the mean ± SD of three determinations.

##### Percentage Moisture Loss

The percentage moisture loss of the polymeric films was determined by computing the difference between the weights determined before and after placing the films in the desiccator (BR65805, Sigma-Aldrich, China) containing anhydrous calcium chloride for a period of three days [35]. Three samples of each casted film were used to check the percentage moisture loss that was computed using Equation (1).

(1) $$\%Moisture\;Lost=\frac{Initial\;Weight-Final\;Weight}{Initial\;Weight}\times 100$$

##### Drug Content

The amount of LOR in the polymeric films was estimated by dissolving the films in ethanol. Films measuring 1 × 1 cm^2^ were dissolved in ethanol (10 mL) and sonicated. The resulting solution obtained was filtered through a 0.45 μm Whatman filter paper. On appropriate dilution with 6.8 pH simulated saliva, the samples were assayed for drug content by measuring the absorbance in a UV spectrophotometer (Shimadzu UV-1900i spectrophotometer, Shimadzu Scientific Instruments, Kyoto, Japan) at 248 nm [36]. The regression equation was obtained from the standard calibration curve of LOR constructed in simulated saliva with ionic strength ranging from 0.12 to 0.17 M at concentrations of 5, 10, 15, 20, 25, and 30 µg/mL [37]. The assay was computed using a calibration curve that had a slope of 0.0266 and an R^2^ value of 0.999. The experiments were performed in triplicate with simulated saliva and the results were expressed as the mean ± SD. The composition of simulated saliva is portrayed in Table 2 [38,39].

##### Disintegration Test

Oral films have to disintegrate rapidly to release their constituent drug quickly. Randomly selected samples from the films (2 × 2 cm^2^) were taken and placed in a USP disintegration test apparatus (Electrolab, ED-2, Mumbai, India) containing 900 mL of simulated saliva at 37 ± 2 °C [40]. The disintegration time (DT) of the rapidly disintegrating film was noted. Six determinations were performed for every formulation, and the results were expressed as the mean ± standard deviation (SD).

#### 2.3.4. Differential Scanning Calorimetry

DSC is an important calorimetric technique used to characterize the solid state of the drug present in the given samples [41,42]. The thermal analysis of LOR, physical mixture, and optimized batch of the film was performed in a differential scanning calorimeter (DSC-60-Shimadzu, Kyoto, Japan). About 10 mg of each sample was taken in a sealed aluminium crucible and evaluated at a heating rate of 10 °C/min in a temperature range of 25–350 °C with purging of nitrogen (50 mL/min) [43]. Data acquisition was undertaken in the temperature range of 80 to 200 °C. The degree of crystallinity (*Xc*) of the physical mixture and optimized film relative to LOR was determined using Equation (2).

(2) $$Xc=\frac{∆Hm}{(1-w).∆H{}^{\circ}m}\times 100$$

where ∆Hm is the experimental heat of fusion (sample), ∆H°m is the heat of fusion of crystalline LOR, and W is the weight fraction of LOR in the sample.

#### 2.3.5. X-ray Diffraction (XRD)

XRD techniques were employed to study the solid state of the drug present in the samples [44]. The diffraction studies were carried out in a powder X-ray diffractometer (LabX XRD-6100, Shimadzu, Japan). X-ray diffractograms of the drug, physical mixture, and optimized batch of the film were recorded individually at a 40 kV voltage and 30 mA current at a scanning speed of 0.5 s per step. The data were acquired in 2θ values ranging from 10 to 32θ to analyze the crystallinity [45]. Crystallinity was determined by comparing the characteristic peak heights in the diffractograms in the samples with those in LOR considered as a reference. The relative degree of crystallinity (RDC) was computed using Equation (3) [46].

(3) $$RDC=\frac{I\;sample}{I\;refer}\times 100$$

where I_refer_ is the intensity of the reference characteristic peak of LOR having the highest intensity, while I_sample_ is the intensity of the sample peak at same 2θ value as the reference.

### 2.4. In Vitro Dissolution Studies

Dissolution studies of the sublingual thin films were performed by the paddle over disc method using a United States Pharmacopeia (USP) dissolution tester (TDT-08L, Electrolab, India) by using 900 mL of simulated saliva as a medium that was maintained at 37 °C ± 0.5 °C [47]. The film was fastened to the disc using acrylate glue, and the paddle was rotated above the film at 50 rpm. About 5 mL samples were withdrawn at 5, 10, 15, 20, 25, 30, 40, and 60 min and replaced with fresh simulated saliva buffers maintained at the same temperature. The absorbance of the samples was measured at 248 nm and assayed for the amount of drug at different time points. The amount of drug dissolved at different time points was determined using the slope of the standard calibration curve as described earlier.

### 2.5. Ex Vivo Mucoadhesion Strength

The modified two-arm balance was employed to assess the mucoadhesive strength of the optimized film [48]. The substrate chosen for evaluating mucoadhesive strength was the goat buccal mucosa. A 2 mm-thick section of goat buccal mucosa was measured and treated with simulated saliva. Following treatment with buffered saline, the mucosa was affixed to a horizontal metal surface using glue. The optimized film, which had a size of 1 × 1 cm^2^, was affixed to the pan of the balance and brought into contact with the mucosa for two minutes. Later, force was applied by sliding the weight on the arm of the balance to the right until the film was detached from the mucosal surface. The force required to detach the film was considered as the mucoadhesive strength and was measured by quantifying the weight in grams [48].

### 2.6. Stability Studies

The optimized film was subjected to short-term stability studies for up to 3 months. The samples were stored with desiccants to control humidity and provide maximum protection from moisture. The temperature (25 °C ± 2 °C) with humidity (60 ± 5%) was adjusted as per ICH guidelines [11]. At the end of the period, the films were examined for any physical changes, disintegration time, and drug release [49].

### 2.7. Statistical Analysis

The results obtained were statistically analyzed using the unpaired Student’s t test in Graphpad Prism version 7.0. A *p* value of <0.05 was considered statistically significant. The data generated were expressed as the mean ± standard deviation (SD).

## 3. Results and Discussion

### 3.1. Formulation of the Sublingual Fast-Dissolving Thin Films

All the sublingual thin films were found to be clear and displayed desirable physicomechanical properties, indicating the suitability of the casting technique to produce sublingual fast-dissolving LOR thin films. LOR is known to exhibit a pH-dependent solubility, as it is more soluble at lower pH values below its pKa [50]. The drug is a weak base that is known to exhibit a pKa of 4.9, indicating that it is nearly 99.6% and 100% ionized at pH 2.5 and 1.2, respectively [51]. Although the drug completely dissolves in the gastric pH, absorption does not happen until it reaches the intestine, invariably resulting in a delayed onset of action when administered perorally. In this context, we planned to exploit the pH-dependent solubility of the drug by formulating a sublingual film of LOR using citric acid as a pH modifier. Citric acid is a weak acid that forms co-amorphous systems that possess better solubility compared to LOR. Thus, casting solutions produced with LOR-CA result in clear polymeric films with a uniform content. On insertion into the sublingual region, a sizable fraction of the LOR remains unionized due to the near-neutral pH prevalent in the sublingual region, promoting drug absorption. Moreover, citric acid, being a saliva-stimulating agent, is able to stimulate the generation of sufficient volume of saliva, thereby improving the dissolution of LOR-CA in the oral cavity. Citric acid has a dual function in the formulation as it forms LOR-CA and also acts as a saliva-stimulating agent that enables fast disintegration of the drug from an orodispersible film in situ [12]. The solubility as well as the dissolution of the co-amorphous LOR-CA system (1:1) were reported to be substantially greater than those of the crystalline and amorphous forms [52]. Furthermore, citric acid (LOR-CA) was reported to enhance the physical stability and bioavailability of LOR [53]. Thus, LOR-CA is likely to enhance drug dissolution in the oral cavity, elicit a faster onset, and improve bioavailability by evading the first-pass effect when administered sublingually. Mitigating pH-dependent drug solubility would also potentially reduce the inter-subject variability in drug absorption between the fasted and fed states [51]. Sodium saccharin and peppermint flavor were used as sweetening and flavoring agents to enhance the acceptability of films.

### 3.2. Evaluation of Sublingual Fast-Dissolving Thin Films

#### 3.2.1. Morphological and Surface Topographical Analysis

**Figure 1:**
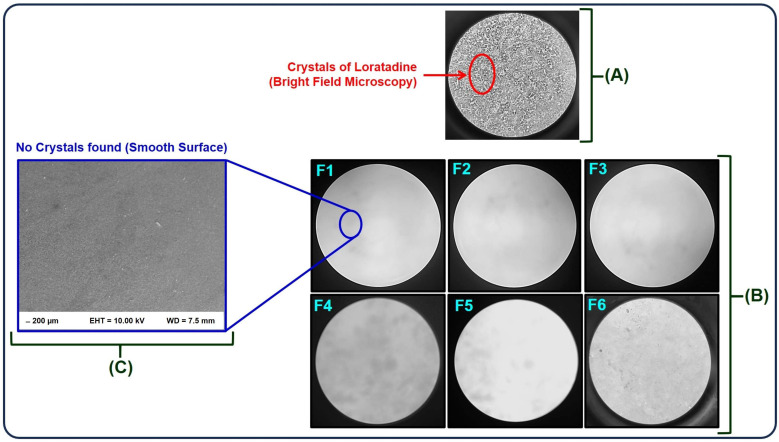
Bright field microscopy image of films composed of LOR (**A**) and LOR-CA (**B**) under a magnification of 100×. SEM analysis report of the F1-casted film (**C**) with a scale bar of 200 µm.

The surface morphology and topographical analysis of sublingual thin films batches are represented in Figure 1. Bright field microscopy (100× magnification) can be employed to assess the crystallinity and surface topography [25]. The films developed without the addition of citric acid lost their clarity and smooth surface. Crystals of LOR were clearly visible when these films were observed under bright field microscopy as indicated in Figure 1A. On the other hand, films of LOR-CA developed using citric acid in a ratio of 1:1 were found to be smooth, clear, and non-tacky as depicted in Figure 1B. The SEM analysis clearly depicted the smooth morphology and topography of the casted polymeric film as shown in Figure 1C [26]. SEM (200 µm scale) also indicated that the surface topography was smooth with no crystalline drug evident on the surface. The absence of the drug crystals could be attributable to the formation of LOR-CA produced by the solvent evaporation method.

#### 3.2.2. Drug–Polymer Compatibility Analysis

**Figure 2:**
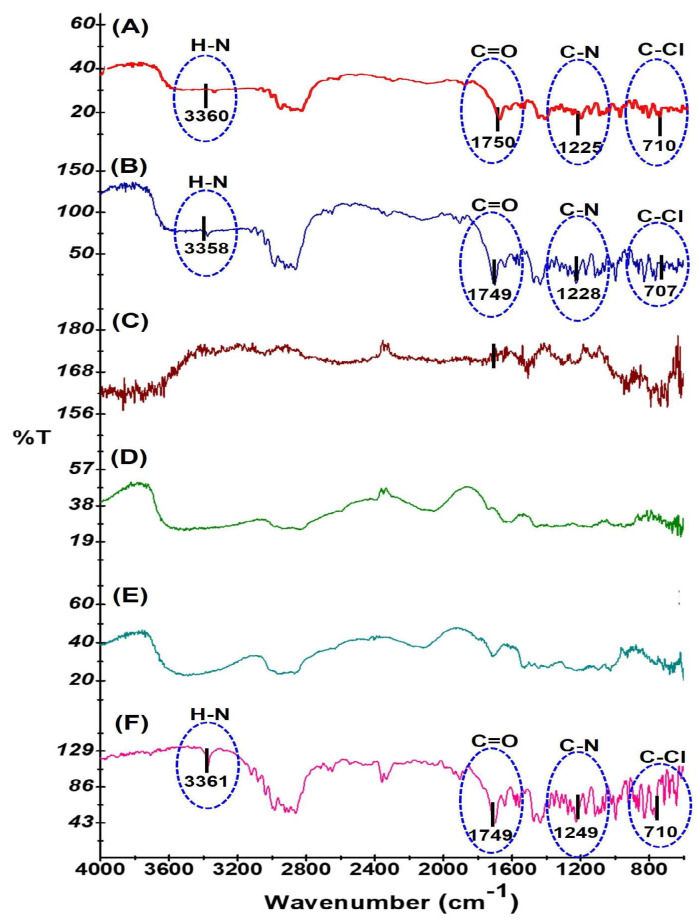
Fourier transform infrared (FTIR) spectra of (**A**) F1, (**B**) physical mixture, (**C**) PVP K30, (**D**) HPC EF, (**E**) HPMC E15, and (**F**) LOR.

The FTIR vibrational spectra of LOR, polymers, a physical mixture, and the optimized film (F1) were recorded and comparisons performed for the physical mixture and F1 with LOR to check for any possible interaction of the polymers. The characteristic vibrational bands of LOR were found to appear at 710, 1749, 3361, and 1226 cm^−1^ in the FTIR spectra of LOR. The vibrational band at 1749 cm^−1^ could be related to C=O stretching, while that observed at 3361 cm^−1^ is likely due to N–H deformation. Similarly, the vibrational band at 710 cm^−1^ can be assigned to C-Cl stretching, whereas the vibrational band at 1226 cm^−1^ may be due to the presence of an aromatic group as a side chain. The results of the spectral analysis were in agreement with the studies that reported FTIR vibrational bands of LOR at 1700, 996.17, 1434, and 1220 cm^−1^ [54]. It was also observed in the present study that a characteristic vibrational band of LOR was found to appear in the same region in the FTIR spectra of the physical mixture (LOR with all polymers) and the film F1 as indicated in Table 3 and Figure 2. All the predominant vibrational bands of LOR were present in the formulation, which confirms that there was no chemical interaction between the drug and the excipients used in the film F1.

#### 3.2.3. Physicochemical Characterizations of Sublingual Fast-Dissolving Thin Films

The films were evaluated to assess the effect of the type and concentrations of polymers and other excipients on the physicomechanical properties of the film including the disintegration time.

##### Weight Variation

The average weight of the film measuring 1 cm^2^ varied from 0.05 ± 0.03 to 0.10 ± 0.03 mg as indicated in Table 4. The films were found to be homogenous in terms of weight, indicating the reproducibility of the film-casting method. Low-viscosity grades of hydrophilic polymers are generally preferred in the fabrication of oral films as they easily disintegrate and release the contents by virtue of their low cross-linking density [55]. LOR sublingual thin films were prepared using different bioadhesive hydrophilic polymers that included HPMC E15, PVP K30, HPC-EF, and polymer blends like HPMC E15-PVP K 30 and HPC-HPMC E15 by the solvent-casting method. The formulation F1 which was composed of lower proportions of HPMC E15 (2.5% *w*/*v*) was found to easily spread on the Petri plate and resulted in films that displayed a uniform thickness and weight.

##### Thickness Measurement

The average thickness of sublingual thin films in each batch varied from 0.23 ± 0.04 to 0.41 ± 0.05 mm, as shown in Table 3. The films were found to exhibit a uniform thickness, indicating the reproducibility of the technique used for the fabrication of the films. The thickness of the films was found to depend on the type and amount of polymer used in the fabrication of the film, as indicated in Table 4. The formulation F1 fabricated using HPMC E15 alone was found to display a thickness of 0.254 ± 0.02 mm. By virtue of its low viscosity, HPMC E 15 could be casted with ease to obtain films that displayed a uniform thickness and weight.

##### Folding Endurance

The FE of F1 to F6 varied from 73.6 ± 5 to 132 ± 3; the values are given in Table 4. The high values of the FE indicated the films produced by the film-casting method were flexible enough. The folding endurance studies indicated that the films produced were flexible with a good mechanical strength. The good folding endurance can be attributed to the incorporation of glycerin as a plasticizer. The flexibility of the film can be attributed to the right proportion of glycerin, which was used to an extent of nearly 60% of the dry weight of the polymer. Glycerin is used in a proportion of 20 to 60% of the dry weight of the polymer in the preparation of orodispersible films [55]. Plasticizers are known to reduce the glass transition temperature of polymers, impart good folding endurance, and improve the overall physicomechanical properties. When HPMC is combined with plasticizers such as glycerin, it achieves good film-forming properties [55]. The good physicomechanical properties of the films justified the selection of the polymers and the plasticizer for the fabrication of sublingual thin films.

##### Tack Test

All the formulations were found to be non-tacky, and the results are given in Table 4. The non tacky nature of the films produced indicated the films produced were dry. Polymeric films that are non-tacky more often result in fast disintegration upon insertion in the sublingual cavity [55].

##### Surface pH

As an extreme range in surface pH is likely to irritate the oral mucosa, surface pH is a critical parameter in the evaluation of orodispersible films. When the film is soaked in water, the surface pH describes the local microenvironment where the film interacts with the surrounding medium. In practice, equilibrating the film in water or another medium may slightly alter the surface pH, depending on the composition of the film (polymers, excipients, etc.) [55]. The surface pH of the films was found to be slightly on the acidic side owing to the presence of citric acid in the composition. Ideally, the orodispersible film needs to possess a close-to-neutral pH to avoid mucosal irritation. The surface pH of all formulations ranged from 6.2 ± 0.1 to 6.5 ± 0.1 (Table 4). As the surface pH was close to neutral and physiological pH (7.35 to 7.45), the films produced would be less prone to irritate the mucosa when inserted into the sublingual region of the oral cavity [34].

##### Percentage Moisture Loss

The percent moisture loss was found to vary from 1.10 ± 0.01 to 2.59 ± 0.07% as shown in Table 4. The minimal moisture loss indicates that the films would be able to maintain their flexibility with aging. The incorporation of the right proportion of glycerin would help to minimize the moisture loss and retain the physicomechanical properties of the films during storage.

##### Drug Content of the Sublingual Fast-Dissolving Thin Films

The uniform drug content of sublingual formulations of different batches displayed uniform content, indicating the reproducibility of the solvent-casting method employed. The drug content values of different batches of the films are depicted in Table 4. The weight and thickness of the films in turn determine the drug content. The LOR content in all sublingual thin films was found to be in the range of 88.02 ± 0.03 to 94.00 ± 1.87%, with a maximum standard deviation within 2.20% indicating a uniform distribution of the drug. The content uniformity can be attributed to the presence of LOR-CA, which displayed better solubility, resulting in clear polymeric films that displayed a uniform drug content.

##### Disintegration Test

The sublingual thin films of LOR were found to quickly disintegrate, where the DT depended on the type and amount of polymer used for casting the films. The DT was found to range from 30.30 ± 0.57 to 125.0 ± 50 s in simulated saliva (Table 4). The DT of the sublingual thin films was found to directly depend on the thickness of the film. The studies indicated that DT was found to be prolonged for thicker films. The molecular weight of the polymer employed was the other key factor that determined the DT. Generally, the DT was found to be quick for thin films that were produced with lower concentrations of polymers. For instance, the formulation F1 comprised of HPMC E 15 that had the least thickness (0.254 ± 0.02 mm) recorded a DT of 30.30 ± 0.57 s which was significantly lower (ANOVA, *p* < 0.0001) compared to the DT displayed by F2 (61.6 ± 2.88 s) which was thicker (0.361 ± 0.007) due to higher amounts of HPMC E15. A similar direct relationship between the thickness of the orodispersible film and the DT was reported earlier [14]. Orodispersible films composed of nanosized LOR were reported to disintegrate in a comparable time span (<60 s) and elicit systemic levels in 60 min [13]. It was also found that DT depended on the type of hydrophilic polymer employed to fabricate the film. The DT of the sublingual thin films in the present study decreased in the following order: HMPC E15 < PVP K30 < HPC-EF as indicated in Table 4. The DT of the film (F1) was significantly lower (ANOVA, *p* < 0.0001) than other formulations casted using PVP K30 and HEC-EF as polymers. The DT could be related to the molecular weight of the hydrophilic polymer, as a quicker DT was observed with low-molecular-weight polymers. It has to be noted that the molecular weight of HPMC E15 (50,000 Daltons) is less than that of PVP K30 (60000 Daltons) and HPC-EF (80,000 Daltons). This could be the likely reason for the significantly shorter DT in the case of F1 compared to other formulations.

#### 3.2.4. Differential Scanning Calorimetry

**Figure 3:**
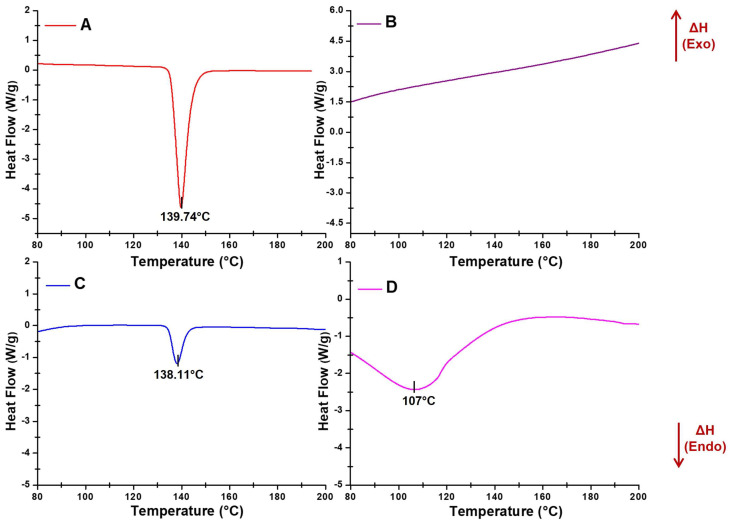
Differential scanning calorimetric thermograms of (**A**) LOR, (**B**) HPMC E15, (**C**) physical mixture, and (**D**) F1.

DSC thermograms for LOR, the physical mixture, and the formulation F1 are captured in Figure 3. A sharp peak of LOR was observed at 139.74 °C with an enthalpy of fusion (∆Hf) value of 79.25 J/g, indicating the crystalline nature of the drug. The melting point observed corresponds to that of LOR, which is reported to be at 139.31 °C [56]. The ∆Hf value observed for LOR was in close agreement with that reported in the literature [57,58]. The endothermic sharp peak of LOR appeared in the physical mixture at 138.11 °C, though the intensity (∆Hf = 24.60 J/g) significantly dropped (ANOVA, *p* < 0.0001) indicating that the crystallinity of LOR dropped to nearly 20%. The thermogram of the physical mixture indicated that nearly 80% of the LOR was found to be soluble in molten HPMC E15. Further, the characteristic peak of LOR was completely transformed to a broad band at 107 °C in the thermogram of the formulation F1 as the degree of crystallinity for the film F1 was further reduced to 3.67%, indicating that LOR was almost in an amorphous form as a solid-solid solution (Figure 3).

#### 3.2.5. X-ray Diffraction

**Figure 4:**
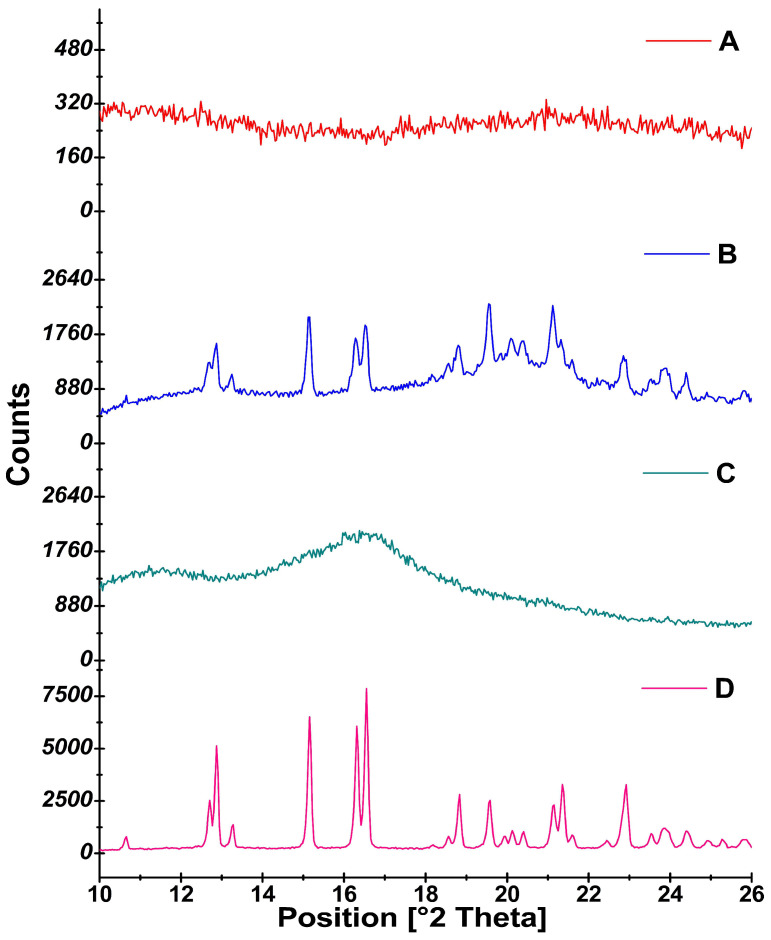
X-ray diffractograms of (**A**) F1, (**B**) physical mixture, (**C**) HPMC E15, and (**D**) LOR.

PXRD was used as a supportive tool to elucidate the solid state of the drug in the optimized film (F1). The XRD spectra of the LOR, physical mixture, and sublingual film are shown in Figure 4. LOR was found to display seven sharp peaks at 12.87°, 15.15°, 16.55°, 18.33°, 19.58°, 21.35°, and 22.92° 2θ values, indicating the crystalline nature of the drug. The peak intensity at 16.55 that displayed the highest intensity of 7856 counts was considered a reference peak. The XRD peaks of LOR were reported to appear at 2θ values of 11°, 12.5°, 14.2°, 16.4°, 19°, 21°, 23°, and 30°, respectively, in the studies undertaken earlier [49]. In the present study, the diffractograms of the physical mixture displayed the characteristic peaks of LOR at 12.87°, 15.15°, 16.55°, 18.33°, 19.58°, 21.11°, and 22.85° 2θ values, indicating that LOR was retained in the crystalline form in the physical mixture. The peak intensity at the 16.55° 2θ value that was considered as a reference peak was found to significantly reduce (ANOVA, *p* < 0.0001) to 1875 counts in the physical mixture. The RDC value of 23.87% indicated that the crystallinity was found to drop by nearly four-fold in the physical mixture. The peak intensity for the formulation F1 at 16.58° was further found to reduce to 231 counts with an RDC value of 2.9 indicating a significant drop (ANOVA, *p* < 0.0001) in crystallinity by nearly 34-fold. Thus, the XRD spectral analysis was in total agreement with the DSC data, indicating the further reduction in crystallinity of the drug in the formulation F1. The near-amorphous nature of LOR in hydrophilic polymers is likely to expedite drug release from the orodispersible films.

### 3.3. In Vitro Dissolution Studies

**Figure 5:**
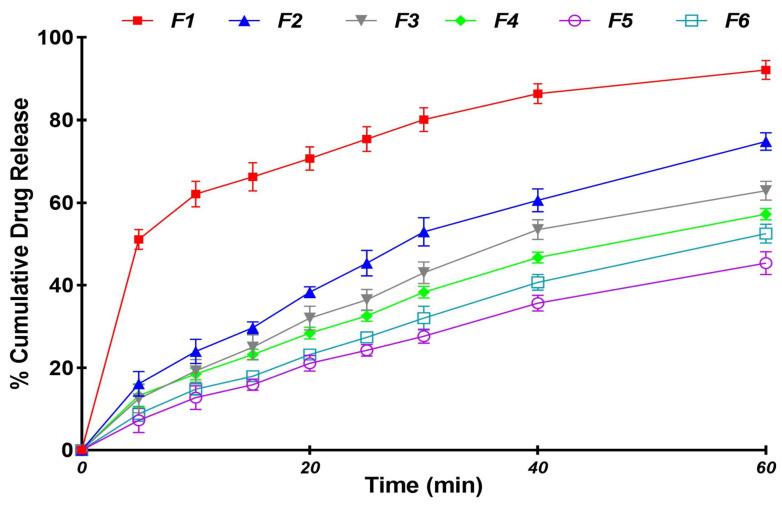
Percent cumulative release profiles of fast-dissolving films of LOR in simulated saliva (6.8 pH). The release at 60 min was significantly higher (ANOVA, *p* < 0.001) for F1 compared to the other formulations.

The in vitro dissolution studies of the sublingual thin films were represented as the percentage cumulative drug release at different time points. The drug release for different batches of formulations was found to range from 45.40 ± 2.80% to 92.1 ± 2.30% in 60 min in simulated saliva. The dissolution profiles of various formulations of sublingual thin films are captured in Figure 5. Generally, the amorphous state of LOR could likely contribute to quicker release from all of the film formulations. More importantly, the type and amount of polymer were found to determine the drug release. Generally, the DT was found to be the rate-determining step that dictated the drug release from the film formulations. The same rank order correlation noted with the DT was found to exist with the amount of drug released at the end of 60 min. Maximum release was observed from films made of HPMC E15, as a release of 92.1 ± 2.30% and 74.80 ± 2.10% was noted for F1 and F2 by 60 min. This was followed by films composed of PVP K30 and, finally, films composed of HPC-EF displayed the least release, indicating the dominant role played by the type of polymer on the drug release. The amount of drug release from the optimized film (F1) that was cast with HPMC E15 was found to be significantly higher (ANOVA, *p* < 0.001) than other film formulations. We observed that films with lower amounts of HPMC E15 disintegrated faster and exhibited quicker release. HPMC E15 is known for its viscosity and ability to form a gel layer upon contact with moisture. When present in lower amounts, it reduced viscosity and gel formation and resulted in a weaker film structure and faster dissolution and drug release, whereas higher HPMC E15 concentrations increased the viscosity and gel strength, slowing water absorption and drug diffusion, thereby delaying film disintegration and drug release [59].

### 3.4. Ex Vivo Mucoadhesion Strength

The bioadhesive films demonstrated a strong tendency to adhere well to the buccal mucosa employed as the substrate. The measured mucoadhesive strength for the optimized film (F1) was determined to be 11.27 ± 0.418 gm/cm^2^. The amount of LOR loaded was 10 mg for all the batches of films that measured 12.6 cm^2^. The sublingual bioadhesive films would be retained well on the sublingual mucosa and therefore are likely to evade drug dissipation into the gastrointestinal tract, thereby minimizing the first-pass effect as well.

### 3.5. Stability Studies

**Figure 6:**
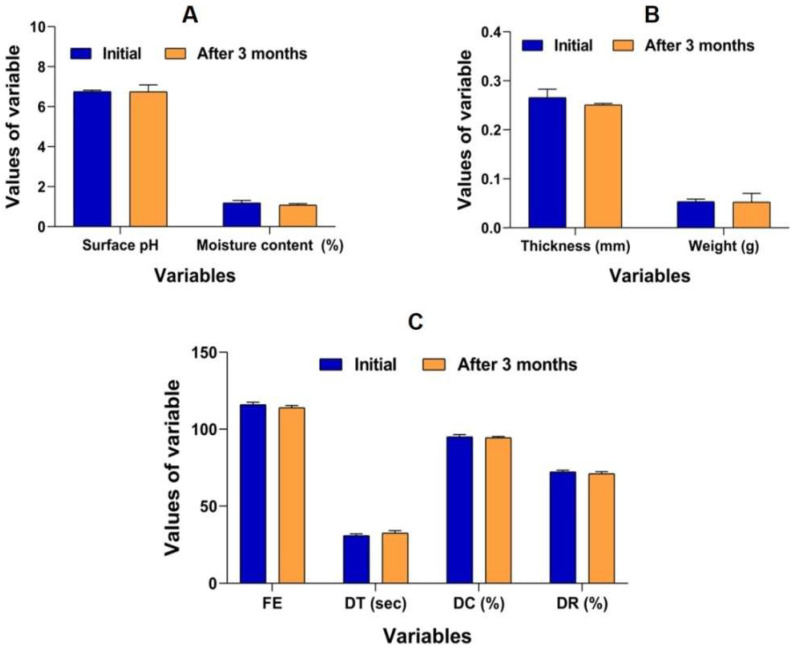
Grouped column representation of stability analysis of optimized film (F1) from the initial day up to the third month. (**A**) surface pH, moisture content, (**B**) thickness, weight, and (**C**) folding endurance (FE), disintegration time (DT), drug content (DC), and drug release (DR).

The optimized film (F1) of LOR was found to demonstrate adequate stability after three months in terms of physicomechanical properties, disintegration, and dissolution as indicated in Table 5 and Figure 6. The stability study performed for a period of three months indicated that there were minute changes in the critical parameters of films in batch F1 (Table 5). The moisture content after three months dropped slightly to 1.03 ± 0.065 compared to the initial moisture content data (1.10 ± 0.125). Furthermore, due to slight changes in the moisture content, we observed a very minor change in the thickness (0.249 ± 0.002) and weight (0.04 ± 0.003) of the optimized film (F1). Overall, there were no significant differences (ANOVA, *p* > 0.05) observed after the completion of the studies, indicating that the films demonstrated adequate stability.

## 4. Conclusions

Sublingual thin films of LOR with the most desirable features were successfully produced by the solvent-casting technique using HPMC E15 as a film former, citric acid as a co-former, and glycerin as a plasticizer. Citric acid is known to form co-amorphous systems with LOR, rendering it more amorphous. Solid-state characterization using differential scanning calorimetry and X-ray diffractometry indicated that LOR-CA was embedded in an amorphous form as a solid-state solution in the films. The sublingual thin films F1 developed with LOR-CA containing a lower amount of HPMC E15 were found to be thin, display uniformity, have a shorter disintegration time, and show maximum release in 60 min. The therapeutic dose of LOR was successfully loaded into a film measuring 12.6 cm^2^. Thus, we were able to successfully improve the drug solubility without the need of a solubilizer that would bypass the first-pass effect and most likely elicit a faster onset, variable bioavailability of LOR, and an increase in patient compliance.

## Figures and Tables

**Table 1:** The composition of casting solutions for preparing sublingual fast-dissolving thin films of loratadine.

Formulation Ingredients	Composition (mg)					
	F1	F2	F3	F4	F5	F6
Loratadine	12.5	12.5	12.5	12.5	12.5	12.5
HPMC E15	250	500	-	250	-	250
PVP K 30	-	-	500	250	-	-
HPC-EF	-	-	-	-	500	500
Glycerin	125	250	250	250	250	250
Citric acid	12.5	12.5	12.5	12.5	12.5	12.5
Sodium saccharin	12.6	12.6	12.6	12.6	12.6	12.6
Peppermint flavor	3	3	3	3	3	3
Ethanol	Up to 10 mL					

**Table 2:** Composition of simulated saliva used for evaluation of polymeric films.

Ingredients	Simulated Saliva
pH 6.8 buffer (mL)	6.8
Tween 20^®^ (µL)	5.6
Xanthan gum (*w*/*v*%)	0.05
Porcine gastric mucin (mg/mL)	10
Porcine pancreatic alpha-amylase (mg/mL	1.0
Deionized water (mL)	up to 100
Final pH adjusted (1M HCl or NaOH)	~6.8

**Table 3:** Fourier transform infrared characteristic vibrational band of LOR, physical mixture, and F1 film.

Characteristic Vibrational Band (cm^−1^)	Functional Groups	Observed Band Positions in Loratadine	Observed Band Positions in Physical Mixture	Observed Band Positions in F1 Film
1700–1780	C=O	1749	1748	1750
3373–3422	H-N	3361	3358	3360
500–730	C-Cl	710	707	708
1230–1050	C-N	1226	1228	1225

**Table 4:** Physicochemical parameters of the sublingual film of loratadine.

Formulations						
Parameters	F1	F2	F3	F4	F5	F6
Thickness (mm)	0.25 ± 0.02	0.36 ± 0.07	0.33 ± 0.04	0.41 ± 0.07	0.31 ± 0.05	0.41 ± 0.05
Weight (mg)	0.05 ± 0.03	0.09 ± 0.02	0.06 ± 0.04	0.1 ± 0.03	0.07 ± 0.02	0.09 ± 0.04
Tack test	Non tacky	Non tacky	Non tacky	Non tacky	Non tacky	Non tacky
Folding endurance	115 ± 1	132 ± 3	123 ± 2	131 ± 1	90.3 ± 1	73.6 ± 5
Surface pH	6.5 ± 0.1	6.5 ± 0.05	6.4 ± 0.05	6.3 ± 0.1	6.2 ± 0.1	6.3 ± 0.1
DT (sec)	30.3 ± 0.57	61.6 ± 2.88	91.6 ± 2.88	105 ± 5.13	93 ± 1.0	125 ± 5.0
Moisture loss (%)	1.10 ± 0.01	2.07 ± 0.06	1.18 ± 0.07	2.19 ± 0.06	1.51 ± 0.15	2.59 ± 0.68
Drug content (%)	94.04 ± 1.87	90.1 ± 0.07	88.1 ± 2.61	89.10 ± 2.2	88.02 ± 0.03	88.7 ± 0.06

DT: Disintegration time.

**Table 5:** Stability data indicating evaluation parameters of the optimized film (F1) on initial measurement (0 months) and after final measurement (3 months).

Parameters	Initial	After 3 Months
Thickness (mm)	0.254 ± 0.016	0.249 ± 0.02
Weight (g)	0.05 ± 0.004	0.04 ± 0.003
Folding endurance	115 ± 0.414	113 ± 0.513
Disintegration time (sec)	30.3 ± 0.163	31.5 ± 0.843
Surface pH	6.7 ± 0.071	6.5 ± 0.5
Moisture content (%)	1.10 ± 0.125	1.03 ± 0.065
Drug content (%)	94.0 ± 0.83	93.8 ± 0.742
%Drug release (25 min)	71.6 ± 0.381	70.14 ± 0.275

## Data Availability

The original contributions presented in the study are included in the article, further inquiries can be directed to the corresponding author.
